# Targeted physiotherapy for patellofemoral joint osteoarthritis: A protocol for a randomised, single-blind controlled trial

**DOI:** 10.1186/1471-2474-9-122

**Published:** 2008-09-16

**Authors:** Kay M Crossley, Bill Vicenzino, Marcus G Pandy, Anthony G Schache, Rana S Hinman

**Affiliations:** 1 Department of Mechanical Engineering, Melbourne School of Engineering, The University of Melbourne, Australia; 2 School of Physiotherapy, The University of Melbourne, Melbourne, Australia; 3 Division of Physiotherapy, School of Health and Rehabilitation Sciences, University of Queensland, Brisbane, Australia; 4 Centre for Health, Exercise and Sports Medicine, School of Physiotherapy, The University of Melbourne, Melbourne, Australia

## Abstract

**Background:**

The patellofemoral joint (PFJ) is one compartment of the knee that is frequently affected by osteoarthritis (OA) and is a potent source of OA symptoms. However, there is a dearth of evidence for compartment-specific treatments for PFJ OA. Therefore, this project aims to evaluate whether a physiotherapy treatment, targeted to the PFJ, results in greater improvements in pain and physical function than a physiotherapy education intervention in people with symptomatic and radiographic PFJ OA.

**Methods:**

90 people with PFJ OA (PFJ-specific history, signs and symptoms and radiographic evidence of PFJ OA) will be recruited from the community and randomly allocated into one of two treatments. A randomised controlled trial adhering to CONSORT guidelines will evaluate the efficacy of physiotherapy (8 individual sessions over 12 weeks, as well as a home exercise program 4 times/week) compared to a physiotherapist-delivered OA education control treatment (8 individual sessions over 12 weeks). Physiotherapy treatment will consist of (i) quadriceps muscle retraining; (ii) quadriceps and hip muscle strengthening; (iii) patellar taping; (iv) manual PFJ and soft tissue mobilisation; and (v) OA education. Resistance and dosage of exercises will be tailored to the participant's functional level and clinical state. Primary outcomes will be evaluated by a blinded examiner at baseline, 12 weeks and 9 months using validated and reliable pain, physical function and perceived global effect scales. All analyses will be conducted on an intention-to-treat basis using linear mixed regression models, including respective baseline scores as a covariate, subjects as a random effect, treatment condition as a fixed factor and the covariate by treatment interaction.

**Conclusion:**

This RCT is targeting PFJ OA, an important sub-group of knee OA patients, with a specifically designed conservative intervention. The project's outcome will influence PFJ OA rehabilitation, with the potential to reduce the personal and societal burden of this increasing public health problem.

**Trial Registration:**

Australia New Zealand Clinical Trials Registry ACTRN12608000288325

## Background

Osteoarthritis (OA) is the leading cause of musculoskeletal pain and disability and is the third leading cause of life-years lost due to disability in Australia, only behind depression and dementia [1]. The annual total cost of arthritic disease in Australia is estimated at $24 billion [2], with the knee joint contributing substantially to this overall cost. The prevalence of OA in people aged over 55 years is 20–26% and rising, with arthritis rates expected to increase by 30% over the next 40 years [2]. The pain and suffering endured by patients as a result of OA decreases their quality of life, with the annual burden of disease costs ($12 billion in Australia) being half the total costs associated with this condition [2]. Pain associated with daily activities such as walking and stair-climbing ultimately leads to profoundly reduced functional independence [2].

The patellofemoral joint (PFJ) is one of the three knee joint compartments. Awareness of its importance in the OA process has been raised by the increasing use of lateral and skyline x-rays in recent times. Research has revealed that PFJ OA is more common than previously thought. In a community-based study of knee OA (N = 218), the frequency of radiographic osteophytes was greater in the PFJ (65% knees) than in the tibiofemoral joint (TFJ) (55% knees) [3]. Furthermore, in people with knee pain (N = 777), the most common compartmental distribution of radiographic OA was a combination of TFJ and PFJ disease (40%), followed by isolated PFJ OA (24%), and isolated TFJ disease (4%) [4]. Within the PFJ, the lateral compartment is more frequently affected by the OA process than the medial [5,6]. Importantly, the presence of baseline PFJ OA predicts structural deterioration in the TFJ compartment over 30 months (OR 2.31, 95% CI 1.37, 3.88) [7].

The PFJ is an important source of symptoms associated with knee OA [8]. Knee pain has been found to be significantly associated with PFJ osteophytes (OR 2.25, 95%CI 1.06, 4.77), but not TFJ osteophytes (OR 1.19, 95% CI 0.46, 3.09) [9], suggesting that the PFJ may be a more important source of knee pain than the TFJ. Hunter et al [10] noted that increased pain and poorer function was associated with reduced cartilage volume in the patella, but not in the femur nor the tibia. Other authors have confirmed the relationship between radiographic PFJ OA and knee pain [11-13].

Management strategies for knee OA have traditionally focussed on alleviating symptoms, primarily using drug therapies or surgery. A meta-analysis of OA trials highlights this, with most trials evaluating drug treatments (60%) or surgical procedures (26%) [14]. OA experts have highlighted the overall dearth of quality evidence to support the use of non-pharmacological interventions such as physiotherapy. Despite this, knee OA clinical guidelines recommend that conservative treatments be included as a first line strategy for the optimal management of the disease [15,16]. Physiotherapy is a conservative intervention, which is non-toxic, inexpensive and promotes physical activity and self management through exercise. Therefore, rigorous randomised clinical trials (RCTs) that evaluate the efficacy of physiotherapy are clearly needed, to better guide clinical decision-making.

Given the heterogeneity of knee OA with regard to aetiology, clinical presentation and natural history, guidelines also recommend the tailoring of knee OA treatments to the location of joint damage in order to optimise treatment outcomes [15,16]. However, most trials of physiotherapy for knee OA have not been targeted to disease subgroups, with participant selection typically based on the presence of non-specific knee pain and radiographic changes anywhere on an anteroposterior radiograph. While a plethora of evidence attests to the benefits of exercise for patients with predominant TFJ OA [17] there is no level I evidence and only one RCT [18] specifically addressing the problem of PFJ OA. The dearth of evidence for a compartment-specific treatment for PFJ OA necessitates our proposed study to establish the efficacy of a compartment-specific physiotherapy treatment using the rigour of a RCT.

While there is little known about the physical impairments associated with PFJ OA, there are several RCTs that have evaluated physical interventions for PFJ pain in younger adults (patellofemoral pain syndrome, or anterior knee pain). We have previously conducted a double blind, placebo-controlled RCT [19], which demonstrated the efficacy of a targeted physiotherapy program for this patient population. The targeted treatment involved (i) quadriceps muscle retraining; (ii) patellar taping; (iii) manual PFJ and soft tissue mobilisation; and (iv) hip muscle retraining. We have recently confirmed the beneficial effects of this targeted physiotherapy approach on pain and physical function in another population of young adults with PFJ pain [20]. Therefore, we are proposing to evaluate a similar, targeted physiotherapy intervention for people with PFJ OA.

This project aims to evaluate whether a physiotherapy treatment, targeted to the PFJ and based on successful treatment for PFJ pain in younger populations, results in greater improvements in pain and physical function than a physiotherapy education intervention in participants with symptomatic and radiographic PFJ OA.

## Methods

### Experimental design

**Figure 1:**
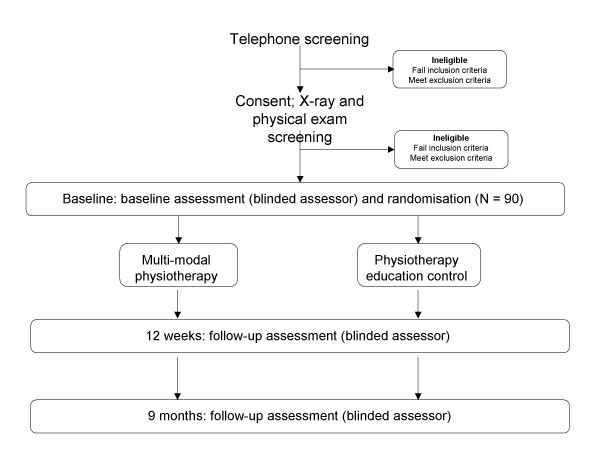
Flow of participants through the randomised controlled trial.

A randomised, single-blind, controlled clinical trial conforming to CONSORT [21] guidelines will be conducted, comparing a multimodal physiotherapy intervention to a physiotherapy education intervention (Figure 1). A Project Investigator will screen for eligibility based on history, clinical and radiographic examination.

Ethical approval has been obtained from the University of Melbourne Human Research Ethics Committee (HREC No. 0721163) and from the Department of Human Services Victoria, Radiation Safety Committee. All participants will provide written informed consent.

### Participants

Ninety people with lateral PFJ OA will be recruited from the community via advertisements, medical practitioners and our own research database. To be included in the study, participants must fulfil the following criteria: (i) aged > 40 years; (ii) anterior- or retro-patellar knee pain aggravated by at least two activities that load the PFJ (eg stair ambulation, squatting and/or rising from sitting); (iii) pain severity ≥ 4 on an 11 point numerical pain scale during aggravating activities; (iv) pain during these activities present on most days during the past month; (v) osteophyte grade ≥ 1 in the lateral PFJ compartment on skyline x-ray [22].

Exclusion criteria will include: (i) concomitant pain from other knee structures, hip or lumbar spine; (ii) current or previous physiotherapy for knee pain (prior 12 months); (iii) contra-indications to the treatments (eg tape allergy); (iv) recent knee injections (prior 3 months); (v) planned lower limb surgery in the following 9 months; (vi) body mass index ≥ 35 kg.m^2^; (vii) medial PFJ OA (osteophytes or joint space narrowing on a skyline x-ray) that is more severe than lateral PFJ OA; (viii) moderate to severe concomitant TFJ OA (Kellgren and Lawrence grade ≥ 3 on an anteroposterior radiograph) [23]; (ix) knee or hip arthroplasty or osteotomy; (xi) physical inability to undertake testing procedures or; (x) inability to understand written and spoken English.

### Sample Size

Treatment efficacy will be evaluated by comparing change on primary outcome measures between groups. We aim to detect the minimum clinically important improvement on these outcomes as reported by Tubach et al [24]. Specifically, a sample of 90 will provide a minimum of 90% power (α = 0.05) to detect a difference in pain on visual analogue scale (VAS) of 19.9 (21.5) mm and a difference in physical function on the Western Ontario MacMaster Universities Osteoarthritis Index (WOMAC) [25] of 9.1 (13.9) normalised units. This sample size also allows for an estimated 10% drop-out rate.

### Procedure

The randomisation schedule (permuted blocks of 8 to 12) will be generated and maintained centrally by one of the investigators (BV), who will not be involved in assessment of participants. The randomisation schedule will be revealed via telephone following baseline assessment. A blinded investigator will perform outcome assessments (Table 1) at baseline, 12 weeks and 9 months, and participants will be instructed not to divulge their group allocation. Security of the blinding system will be evaluated to ensure integrity.

**Table 1:** Outcome measures used in the randomised controlled trial

**Primary Outcome**	**Measurement**
Usual pain on movement in the previous week	0–100 mm visual analogue scale (VAS) with terminal descriptors: 0 = no pain; 10 = maximal pain
Usual pain during nominated aggravating activity in the previous week	0–100 mm visual analogue scale (VAS) with terminal descriptors: 0 = no pain; 10 = maximal pain
Self reported difficulty with physical function	Physical Function subscale of the Western and Onatario MacMasters University (WOMAC) Osteoarthritis Index (Likert version)

**Secondary Outcomes**	**Measurement**

*Symptoms*	
Pain and stiffness	Pain and Stiffness subscales of the WOMAC
Perceived global effect score	5 point ordinal scale (1-much improved; 2-improved. 3-same; 4-worse; 5-much worse)
Sports and recreation function	Sports and recreation function dimension of the Knee Injury and Osteoarthritis Score (KOOS)
Symptoms	Symptoms dimension of the KOOS
Knee related quality-of-life	Knee related quality-of-life dimension of the KOOS
*Function*	
Lower extremity functional performance	One-leg rise test – maximum number of one-leg rises from sitting on a 0.48 m stool
Stair ambulation performance	Timed stair ascent and descent
Standing balance	Step test – number of times can step foot up and down off 15 cm step in 15 s

**Other Outcomes**	**Measurement**

Physical activity levels	Physical Activity Scale for the Elderly (PASE)
Adherence (physiotherapy group only)	Number of physiotherapy visits Completion of home exercises via log-book
Knee-related medication use	Log-book
Adverse effects	Log-book and open probe questioning

### Outcome assessment

Age, gender, duration of knee OA symptoms, previous treatment, surgery and medication use for knee OA will be obtained at the baseline assessment.

#### Primary outcome measures: Pain and physical function

Overall average *knee pain *in the previous week on movement and during an aggravating activity nominated by the participant will be self-assessed with a 0–100 mm horizontal visual analogue scale (VAS) with terminal descriptors of (0 = no pain; 10 = maximal pain). Self-reported difficulty with *physical function *will be assessed using the physical function subscale of the Likert version of the WOMAC [25]. This disease-specific measure is reliable, valid and responsive and comprises 17 items, using a 5-point scale to score each, where higher scores indicate worse symptoms.

#### Secondary outcome measures

*Pain and stiffness *will be assessed using the relevant subscales of the WOMAC [25]. Participants will rate their perceived overall change in symptoms following treatment on a 5 point ordinal scale: 1-much improved, 2-improved, 3-no change, 4-worse, 5-much worse, giving a *perceived global effect score*. *Sports and recreation function, symptoms *and *knee-related quality-of-life *will be assessed using the relevant dimensions of the Knee Injury and Osteoarthritis Outcome Score (KOOS) [26].

*Objective measures of function *will include the one-leg rise test, a timed stair ambulation test and the step-test. The one-leg rise test is the maximum number of one-leg rises the participant can perform from sitting on a stool. The participant must hold their non-test leg out straight and cannot use their arms for assistance. The number of rises that the participant can complete will be recorded. This test is a measure of lower extremity functional performance that has been found to predict the development of radiographic knee OA in middle aged people with chronic knee pain [27]. The timed stair ambulation task involves the participant ascending and descending a set of nine standard steps at their usual pace and the total time taken recorded, with longer time taken indicating poorer physical function [28]. The step-test is a functional, dynamic test of standing balance, where the participants stands on one leg in front of a 15 cm step, and places the opposite foot on and off the step as quickly as possible over 15 seconds. The total number of successful steps are recorded, with higher scores indicating better balance [29].

#### Other measures

*Disease severity *of the TFJ from weight bearing anteroposterior knee x-rays taken at screening will be determined using the Kellgren and Lawrence grading system [23] where 0 = normal; 1 = possible osteophytes; 2 = minimal osteophytes and possible joint space narrowing; 3 = moderate osteophytes, some narrowing and possible sclerosis and; 4 = large osteophytes, definite narrowing and severe sclerosis. PFJ OA will be assessed from a skyline x-ray using a radiographic atlas [22]. The medial and lateral PFJ compartments will each be scored separately for the presence of osteophytes and joint space narrowing where 0 = normal; 1 = mild or 1–33% abnormal; 2 = moderate or 34–66% abnormal and; 3 = severe or 67–100% abnormal. *Co-interventions, adherence and adverse effects will also be recorded*. Participants will be asked to refrain from other forms of OA treatment, but stable drug doses will be permitted. Physiotherapists will record attendance, details of treatment progression (physiotherapy group) and adverse events. Participants will record adherence with home exercises (physiotherapy group), adverse events and any co-interventions, including knee-related medication use in a log-book.

### Interventions

Each participant will be treated by an experienced and registered physiotherapist. Treating practitioners will be trained and proficient in both of the interventions (physiotherapy and education control). Each treatment will be delivered in 8 sessions over 12 weeks (once per week for four weeks, then once every two weeks for 8 weeks). Reasonable costs associated with treatments will be met by the project.

#### Physiotherapy Treatment

The physiotherapy treatment will be similar to that employed in our previous RCTs for patellofemoral pain in younger people [19,20]. Treatment will consist of (i) functional retraining exercises for the quadriceps muscle; (ii) quadriceps and hip muscle strengthening; (iii) patellar taping; (iv) manual PFJ and soft tissue mobilisation; and (v) OA education. The treatment will be tailored according to each participant's clinical presentation (eg strength, pain severity, swelling) as well as the presence of co-morbidities (eg back and hip pain or pathology), and will be progressed based on individual response to exercise load, thus optimising treatment effects. Exercises will be taught and supervised by the physiotherapist during each visit. A home exercise program will be prescribed, to be performed independently at home 4 times per week. An exercise manual for participants will be produced, with clear instructions and diagrams to ensure correct and safe performance of exercise. Specific aspects of the treatment are outlined in Table 2 and will include:

**Table 2:** Physiotherapy treatment components

**Functional retraining exercises^† ^performed four times/week – participants perform a contraction of medial quadriceps in two of the following functional activities**	
- sitting (isometric)	
- sit-stand	
- step up	
- single leg squat	
**Quadriceps muscle strengthening^† ^performed four times/week – participants complete one exercise in each of the following**	
- inner range (open kinetic chain)	
- mid range (open kinetic chain)	
- weight-bearing (wall squat)	
**Hip abduction strengthening^† ^performed four times/week**	
- sidelying hip abduction	
**Patellar taping**	
- combination of tilt, medial glide and fat pad unloading – tape will be applied by the physiotherapist at each visit, worn continuously for one week and then removed	-
**Patellofemoral and soft-tissue mobilisation**	
- mobilisation of the patella (medial glides) performed by the physiotherapists	
- massage to the painful and tight soft tissue structures, performed by the physiotherapist	

^†^Exact exercise and its number of repetitions will be determined by the physiotherapist from a schedule of permissible exercises based on each participant's clinical presentation (eg strength, pain severity, swelling), presence of comorbidities (eg back and hip pain or pathology) and will be progressed based on individual response to exercise load

(i) Functional retraining exercises for the quadriceps muscle. The muscle retraining is designed to enhance the co-ordination (magnitude and onset timing) of the medial quadriceps, relative to the lateral utilising biofeedback within the sessions. In order to accommodate a patient group with heterogeneous symptoms, the functional retraining exercises may be performed statically and/or dynamically during various functional activities (eg step up, step down, sit to stand).

(ii) Quadriceps and hip abduction strengthening. The exercise selection will be based on baseline capacity of the individual and then progressed, based on response to exercise load, thus maximising the training effects. Resistance will be provided by weights, rubber tubing and/or body weight.

(iii) Patellar taping to reduce pain using the same standardised protocol as per our previous knee OA research [30,31]. The tape will be applied by the physiotherapist at each visit, worn continuously for one week and then removed.

(iv) Manual PFJ and soft tissue mobilisation, comprising medial patellar glides and massage to the lateral soft tissue structures, performed by the physiotherapist.

(v) OA education covering topics such as exercise, diet, weight loss etc.

Following cessation of supervised physiotherapy sessions at 12 weeks, participants will be instructed to continue with a home exercise program. Adherence to the program will be monitored from the diary recordings of exercise completions.

#### Physiotherapy Education Control

In order to control for the psychosocial contact inherent with the physiotherapy treatment, participants allocated to the control group will attend individualised OA education sessions covering topics such as exercise, diet, weight loss, etc, provided by the physiotherapist with the same frequency as the physiotherapy sessions.

### Data quality and management

Strategies employed to ensure data quality include: (i) training of assessors and physiotherapists; (ii) assessment of procedural quality; (iii) random checks by investigators of adherence to study protocols; and (iv) random checks of forms for completeness and data for accuracy. All analyses will be conducted on an intention-to-treat basis. The primary outcomes measured at 12 weeks and 9 months will be analysed using linear mixed regression models, including their respective baseline scores as a covariate, subjects as a random effect, treatment condition as a fixed factor and the covariate by treatment interaction. Participant characteristics (eg; gender, radiographic severity of TFJ and PFJ OA) will also be included as covariates. Regression diagnostics will be used to check for normality of the measures and homogeneity of variance, where appropriate. Comparisons between group means will be performed using Bonferroni or Newman Keuls range tests. An alpha level of 0.05 will be used. Calculation of the number needed to treat index will be performed to facilitate the development of clinical guidelines.

## Discussion and Conclusion

PFJ OA is emerging as a distinct clinical entity that is common, is associated with considerable pain and disability, and is an important and novel area of research, since little is known about the optimal management of this condition. This study uses a single-blind RCT design to investigate whether a multimodal physiotherapy treatment, targeted to the PFJ, is more effective in reducing pain and improving physical function than a physiotherapy education control intervention in people with PFJ OA. As a secondary aim, it will evaluate whether the targeted physiotherapy treatment results in greater perceived improvement, self-reported stiffness, pain, sport and recreational function, symptoms and knee-related quality of life, as well as performance on functionally relevant tasks (one-leg rises, timed stair ambulation, and step-test) than the physiotherapy education control intervention.

In contrast to OA primarily affecting the TFJ, comparatively little known about the features or impairments associated with OA of the PFJ, and hence designing a targeted intervention is challenging. Thus, we have chosen to investigate a physiotherapy intervention that is largely based on a program that we have previously found to be successful in younger people with PFJ pain (patellofemoral pain syndrome) [19,20]. Components of this targeted intervention include: (i) functional retraining of the quadriceps muscle; (ii) quadriceps and hip muscle strengthening; (iii) patellar taping; (iv) manual PFJ and soft tissue mobilisation; and (v) OA education. This intervention is currently considered to be "best-practice" in the management of PFJ pain, and is increasingly being employed clinically in the management of people with PFJ OA.

An impairment that has been the subject of recent evaluation in participants with generalised knee OA is patellar malalignment. Patellar malalignment is typically exhibited as lateral patellar tilt, displacement or subluxation and may be important in PFJ OA by reducing and lateralising the PFJ contact area [32], thus increasing stress in this compartment. In people with knee OA, PFJ malalignment has been shown to be associated with indices of OA (joint space narrowing and loss of cartilage thickness) [33,34] as well as progression of OA (joint space narrowing) [35] in the PFJ compartment and increased functional impairment [36]. Thus, PFJ malalignment is a key feature of PFJ OA that could be amenable to a targeted intervention such as physiotherapy. This supports the inclusion of patellar tape in our targeted treatment, since it has the potential to reduce patellar malalignment [37-39] and we have already shown that patellar tape can reduce knee pain in generalised knee OA populations [30,31]. Other treatment modalities (eg PFJ and soft tissue mobilisations), may assist in the treatment of PFJ pain and malalignment in this patient population.

The balance of medial and lateral quadriceps activity is essential to maintain PFJ alignment. Experimental studies confirm that reduced or delayed medial quadriceps activity (relative to the lateral quadriceps) increases lateral patellar malalignment, leading to areas of heightened contact stress across the lateral PFJ compartment [40,41]. Thus, the balance of muscle activation between the medial and lateral quadriceps may be important in PFJ disease. In our studies of younger people with PFJ pain [42,43], we have observed a temporal delay in medial quadriceps activity. Thus, it is likely that individuals with PFJ OA may require a specific retraining program designed to restore balanced quadriceps activity.

While the role of hip muscle function in PFJ OA has not been investigated, there is increasing evidence that hip muscle function is impaired (reduced strength [44], delayed hip muscle activity [45]; and altered hip movements during ambulation [46]) in other PFJ conditions. These studies indicate that hip abduction is particularly relevant in patients with PFJ pain and hence, this study is focusing on strengthening hip abduction. Furthermore, the inclusion of a hip abduction strengthening program in this study reflects contemporary clinical practice.

While the main goal of treatment for OA is to reduce pain and disability, it is not known how non-pharmacological interventions achieve this goal; such is the complex multi-factorial nature of OA pain. Our intervention is based on reversing the compartment-specific impairments *likely *to be associated with PFJ OA. Furthermore, this intervention builds on our previous studies, which have established that: (i) taping the patella medially reduces pain and disability associated with non-specific knee OA [30,31,47] and may reduce PFJ malalignment [48] and (ii) a quadriceps retraining program can reduce pain and disability, as well as restore quadriceps muscle activation patterns in younger people with PFJ pain [19,49,50]. Our unique RCT is targeting PFJ OA, an important sub-group of knee OA, with a specifically designed intervention. The project's outcome will influence knee OA rehabilitation, thus reducing the personal and societal burden of this increasing public health problem.

## Competing interests

The authors declare that they have no competing interests.

## Authors' contributions

KC, RH, BV, MP, AS attained the project funding. KC, RH, BV conceived and designed the trial protocol. All authors contributed to the manuscript and have read and approved the final manuscript.

## Pre-publication history

The pre-publication history for this paper can be accessed here:


